# Combined in vivo metabolic effects of quetiapine and methadone in brain and blood of rats

**DOI:** 10.1007/s00204-023-03620-2

**Published:** 2023-10-23

**Authors:** Laura Smedegaard Heisel, Freja Drost Andersen, Sâmia Joca, Lambert Kristiansen Sørensen, Ulf Simonsen, Jørgen Bo Hasselstrøm, Charlotte Uggerhøj Andersen, Kirstine Lykke Nielsen

**Affiliations:** 1 Department of Forensic Medicine, Aarhus University, Palle Juul-Jensens Boulevard 99, DK-8200 Aarhus N, Denmark; 2 Department of Biomedicine, Aarhus University, Høegh-Guldbergs Gade 10, DK-8000 Aarhus C, Denmark; 3 Department of Clinical Pharmacology, Aarhus University Hospital, Palle Juul-Jensens, Boulevard 99, DK-8200 Aarhus N, Denmark

**Keywords:** Quetiapine, Methadone, Metabolomics, LC-MS, Drug metabolites

## Abstract

Changes in pharmacokinetics and endogenous metabolites may underlie additive biological effects of concomitant use of antipsychotics and opioids. In this study, we employed untargeted metabolomics analysis and targeted analysis to examine the changes in drug metabolites and endogenous metabolites in the prefrontal cortex (PFC), midbrain, and blood of rats following acute co-administration of quetiapine and methadone. Rats were divided into four groups and received cumulative increasing doses of quetiapine (QTP), methadone (MTD), quetiapine + methadone (QTP + MTD), or vehicle (control). All samples were analyzed using liquid chromatography-mass spectrometry (LC–MS). Our findings revealed increased levels of the quetiapine metabolites: Norquetiapine, O-dealkylquetiapine, 7-hydroxyquetiapine, and quetiapine sulfoxide, in the blood and brain when methadone was present. Our study also demonstrated a decrease in methadone and its metabolite 2-ethylidene-1,5-dimethyl-3,3-diphenylpyrrolidine (EDDP) in the rat brain when quetiapine was present. Despite these findings, there were only small differences in the levels of 225–296 measured endogenous metabolites due to co-administration compared to single administrations. For example, N-methylglutamic acid, glutaric acid, p-hydroxyphenyllactic acid, and corticosterone levels were significantly decreased in the brain of rats treated with both compounds. Accumulation of serotonin in the midbrain was additionally observed in the MTD group, but not in the QTP + MTD group. In conclusion, this study in rats suggests a few but important additive metabolic effects when quetiapine and methadone are co-administered.

**Supplementary Information:**

The online version contains supplementary material available at 10.1007/s00204-023-03620-2.

## Introduction

Quetiapine is one of the most widely used antipsychotics. Concomitant use or abuse of quetiapine and the opioid methadone and co-occurrence of the two drugs in fatal opioid poisonings seems to increase in Denmark (Andersen et al. 2021). Due to the sedative and cardiovascular effects of both drugs, they may theoretically have additive effects (Andersen et al. 2021). However, investigation of the combination of quetiapine with opioids, like methadone, is sparse. Quetiapine is used to treat mental illnesses like schizophrenia, bipolar disorders, major depressive disorders, and anxiety and exerts its effects through several receptors, including serotonin, dopamine, histamine, and adrenoceptors (Dev and Raniwalla 2000; Nemeroff et al. 2002). Affinity for histamine H_1_ receptors may induce sedative and tranquilizing effects, which is why it is also used as a drug of abuse (Andersen et al. 2022). Metabolic biotransformation of quetiapine involves cytochromes P450 (CYPs) and uridine 5’-diphospho-glucuronosyltransferases (UGTs). Among the most dominant or pharmacologically active, CYP3A4 and, to a minor extent, CYP3A5 give rise to norquetiapine, quetiapine sulfoxide, and O-dealkylquetiapine in humans. CYP2D6 is additionally known to give rise to 7-hydroxyquetiapine and 7-hydroxynorquetiapine as well as norquetiapine sulfoxide and quetiapine M1 in humans (Le Daré et al. 2020).

Among opioids, methadone has analgesic, euphoric, and sedative effects when binding as an agonist to µ-, κ-, and δ-opioid receptors and as an antagonist to the N-methyl-d-aspartate receptor (Dinis-Oliveira 2016). Methadone poisoning is a common cause of death, as it may cause respiratory depression and heavy sedation (Simonsen et al. 2020). The risk of death from opioid overdose has been shown to increase when combined with other sedatives like benzodiazepines (Sun et al. 2017). Metabolism of methadone occurs primarily through CYP3A4 and CYP2B6. The major metabolites are 2-ethylidene-1,5-dimethyl-3,3-diphenylpyrrolidine (EDDP) and 2-ethyl-5-methyl-3,3-diphenylpyraline (EMDP) (Kharasch 2017).

Uehlinger et al. (2007) investigated plasma concentrations of methadone in tolerant patients undergoing methadone maintenance treatment and starting antipsychotic therapy with quetiapine. They found quetiapine to increase (*R*)-methadone plasma concentrations following at least seven days of quetiapine administration. They suggested that quetiapine interacts with CYP2D6 metabolism and/or the P-glycoprotein (P-GP) transporter system to transport methadone out of the cells (Uehlinger et al. 2007). The P-GP transporter is an important structure in the blood–brain barrier and therefore plays an important role in limiting drug access to the central nervous system, and quetiapine has shown a relatively high affinity for P-GP (Boulton et al. 2002).

Another study by Andersen et al. (2022) examined whether quetiapine in combination with methadone or morphine would increase the risk of fatal opioid poisoning owing to its additive inhibitory effects on the central nervous system. The study included data from autopsy cases of fatal poisonings with methadone and/or morphine and data from a survey of drug users, and a behavioral sedation study in rats. The study showed no additive sedative effects of quetiapine and methadone compared with methadone alone in rats. Furthermore, another study in rats showed no additive effects of methadone and quetiapine on respiratory frequency and hemodynamic parameters, but on the other hand, the two drugs seemed to synergistically lower body temperature (Andersen et al. 2023). Because of the widespread mechanisms of action of the two drugs, multiple different mechanisms can be affected, which cannot readily be obtained by measurements of behavioral or physiological parameters. Therefore, in the present study, we used an untargeted metabolomics strategy and targeted analysis to investigate changes in drug metabolite levels and the impact on endogenous metabolites in prefrontal cortex (PFC), midbrain, and blood collected from rats. Our aim was to obtain a broad view of possible synergistic metabolic changes in the brain and blood following acute intoxication with co-administration of quetiapine and methadone. Drug metabolite levels were additionally investigated in blood from humans driving under the influence of drugs (DUID) shown to be positive for both quetiapine and methadone.

## Materials and methods

### Materials for the animal study

Quetiapine fumarate 30 mg/mL was purchased from Sigma-Aldrich (USA) and was dissolved in saline with 15% β-CD by mixing (2500 rps), ultrasound, and heating at 37 °C. Methadone hydrochloride 10 mg/mL was purchased from Skanderborg Pharmacy (Denmark). Saline with 15% sulfobutyl ether beta-cyclodextrin sodium (β-CD) was purchased from Glentham Life Sciences Ltd. (UK).

### Chemicals

Quetiapine fumarate certified reference material was purchased from Sigma-Aldrich (Germany). Norquetiapine certified reference material was purchased from Cerilliant Corporation (USA). O-dealkylquetiapine was purchased from Molcan Corporation (Canada). Quetiapine-D_8_ fumarate and Norquetiapine-D_8_ were purchased from Toronto Research Chemicals Inc. (Canada). A mass spectrometry metabolite library of standards was purchased from IROA Technologies (USA). All other chemical (metabolite) standards were purchased from Sigma-Aldrich (Germany). Acetonitrile (MeCN) (LC–MS grade), methanol (MeOH) (LC–MS grade), and formic acid (FA) were acquired from Merck (Germany). Purified water was prepared using a Milli-Q IQ 7000.

### Animal study

The animal study has previously been described by (Andersen et al. 2022). In short, male Flinders Resistant Line (FRL) rats weighing 300–400 g were bred and housed in a controlled environment at the Translational Neuropsychiatry Unit, Aarhus University, Denmark (TNU). Rats were divided by randomization into four groups receiving cumulative increasing doses of either methadone (2.5, 10, and 15 mg/kg, MTD), quetiapine (3, 10, and 30 mg/kg, QTP), quetiapine + methadone (3 + 2.5, 10 + 10, and 30 + 15 mg/kg, QTP + MTD), or saline with 15% β-CD (control). The observers were blinded to the treatment. The doses were injected intraperitoneal (i.p.) with 30 min between each dose. Four rats were injected and examined at a time. Thirty minutes after the last injection, the four rats were sacrificed successively one by one by decapitation, meaning that the time from injection to decapitation could vary from approximately 30–60 min. PFC and midbrain were dissected on ice after decapitation, and whole blood was collected in tubes containing a fluoride oxalate mixture. All samples were immediately frozen and kept at – 80 ℃ until analysis.

### Human blood samples

Whole blood samples were collected from drivers suspected of driving under the influence of drugs (DUID) by the Danish police in western Denmark. The tubes contained a fluoride oxalate or a fluoride citrate mixture. Samples were subsequently sent under cooled conditions to our department, where they were frozen immediately at – 80 ℃. A total of 114 samples in which an initial commissioned LC–MS screening showed both quetiapine and methadone in the blood were selected for anonymous quantification of quetiapine, norquetiapine, and O-dealkylquetiapine.

### Untargeted UPLC-HR-QTOF-MS metabolomics

#### Preparation of blood samples for UPLC-HR-QTOF-MS

Whole blood samples from the rats were allowed to thaw on ice before 300 µL was aliquoted into new tubes, and 600 µL of ice-cold 80% MeOH was added. Each mixture was shaken at 1000 RPM for 5 min followed by incubation on ice for 10 min. The supernatants were collected in separate tubes following centrifugation at 5000 × g for 10 min. The samples were extracted once again by adding 600 µL of ice-cold 80% MeOH to the pellets to increase extraction efficiency. The pellet mixtures were mixed at 1000 RPM for 5 min, incubated on ice for 10 min, and centrifuged at 5000 × g for 10 min. The supernatants from this second extraction were collected and added to the tubes with the supernatants from the first extraction. The supernatants were evaporated to dryness in a SpeedVac at 35 °C. Before LC–MS analysis, the dried samples were re-suspended in 200 µL of water with 0.1% FA. Quality control (QC) samples were made by pooling an equal amount of all samples into one vial.

#### Preparation of brain samples for UPLC-HR-QTOF-MS

Midbrain and PFC samples from the rats were separately weighed (approx. 20 mg) into 2-mL lysing tubes with ceramic beads for soft tissue homogenization, added 2-mL ice-cold 80% MeOH, and homogenized using a Precellys Evolution tissue homogenizer (Bertin Technologies, France) using a soft tissue program (speed 5800 rpm, cycle 2 × 15 s, pause 30 s at 4 ℃). The samples were incubated on ice for 10 min and centrifuged at 10,000 × g for 10 min. The supernatants were collected into separate tubes. The samples were extracted once again to increase extraction efficiency by adding 1 mL of ice-cold 80% MeOH to the pellets. The pellet mixtures were homogenized by mixing 15 s, pausing 30 s, and mixing again for 15 s at 4 ℃ in a homogenizer, followed by incubation on ice for 10 min. Following centrifugation at 10,000 × g for 10 min, the supernatants were collected and added to the tubes with the supernatants from the first extraction. The supernatants were evaporated to dryness in a SpeedVac at 35 ℃ and re-suspended in water with 0.1% FA (100 µL/3.5 mg tissue) before LC–MS analysis. QC samples were made by pooling an equal amount of all samples into one vial for each tissue type.

#### Untargeted UPLC-HR-QTOF-MS metabolomics analysis

All samples for untargeted metabolomics analysis were analyzed using an ACQUITY I-Class UPLC system (Waters Corporation, Milford, MA, USA) coupled to a Bruker maXis Impact QTOF mass spectrometer (Bruker Daltonics, Bremen, Germany) operated in positive and negative electrospray ionization (ESI) mode.

Chromatographic separation was performed using an ACQUITY UPLC HSS T3 C18 column (2.1 mm × 100 mm, 1.8 µm, Waters) with a gradient elution. Mobile phase A was water with 0.1% FA and mobile phase B was MeCN with 0.1% FA. The column temperature was 50 °C, and the flow rate was 0.4 mL/min. The gradient was as follows: kept at 0% B (0–2 min), then linear increases going from 0 to 40% B (2–6 min), 40–60% B (6–6.5 min), 60 to 88% B (6.5–11 min), 88 to 100% B (11–11.5 min), kept at 100% B (11.5–17 min), linear decrease from 100 to 0% B (17–18 min), and kept at 0% B (18–21 min) for column equilibration. The injection volume was 6 µL and 10 µL for the midbrain in positive and negative ESI, respectively. For PFC, the injection volumes were 4 µL and 10 µL in positive and negative ESI, respectively. The injection volume of blood was 6 µL in both ionization modes. The sample temperature was 6 ℃.

MS scans were acquired in full scan mode at a sampling rate of 4 Hz using a mass range of 50–1000 *m/z*. The nebulizing gas pressure was 1.2 bar, and the capillary voltage was 4.0 kV in positive ESI and 2.5 kV in negative ESI. The drying gas flow was 8.0 L/min at a temperature of 220 °C. MS/MS scans in data-dependent acquisition (DDA) mode (auto-MS/MS) were acquired under similar conditions but with collision energies of 10, 20, or 30 eV at a sampling rate of 10 Hz. Internal calibration was performed at the end of each run using sodium formate in both ionization modes.

Each matrix was analyzed as a single batch. An instrument control sample containing 37 standard compounds was analyzed in the beginning and the end of each batch for quality assessment of instrument performance. The system was additionally equilibrated with four injections of the QC sample in the beginning of each batch. Data acquisition of the samples within a batch was randomized with QC sample injections in between every six or seven samples for quality assessment of instrument performance during the batch. Good instrument performance was achieved if all the QC measurements had identical retention times and intensities of the peaks. If this was not achieved, the batch was reanalyzed. The QC samples were additionally subject to MS/MS fragmentation for metabolite annotation (see the section about *metabolite annotation*). The QC samples were a pool of all the samples for each tissue type and should, thus, contain all the metabolites present in all the samples.

#### Pre-processing of metabolomics data

The mass spectrometry data from UPLC-HR-QTOF were converted to *mzML*. file format using msConvert from ProteoWizard (http://proteowizard.sourceforge.net). The *mzML*. files were pre-processed using XCMS version 3.18.0 (Smith et al. 2006) in R (version 4.2.1). The centWave algorithm (Tautenhahn et al. 2008) was used for peak picking with a resolution of 12 ppm and a signal-to-noise ratio set to 6. The OBI-Warp algorithm (Prince and Marcotte 2006) was used for retention time correction, and gap filling was conducted to recover missing signals in the raw data. Features had to be present in at least 70% of the samples within a sample group (QTP, MTD, QTP + MTD, and control) to be considered for further analysis. Isotopes, ion source fragments, and adducts were annotated using CAMERA version 1.52.0 (Kuhl et al. 2012). Features with coefficients of variation (CV) above 30% of the QC samples were removed from the data table, and each sample was normalized to the total peak intensity of the chromatogram. A tabulated metabolite feature list with the normalized data was retrieved for each sample matrix (blood, midbrain, and PFC).

#### Metabolite annotation

Features were annotated using metID (Shen et al. 2021) with the use of the following databases: massbank, mona, NIST, msdatabase, orbitrap, hmdb, and our own in-house database. Our in-house database include data from authentic reference standards (*m/z*, retention time, and MS/MS) measured on our own system. The database includes around 500 metabolites. Annotations were based on MS/MS measurements of the QC samples. Metabolites of interest were manually quality assured. Annotations from the rat study are in dataset 1.

### Quantification of quetiapine, norquetiapine and O-dealkylquetiapine by LC–MS/MS

#### Equipment and materials for LC–MS/MS analysis

The liquid chromatography system was an Exion UHPLC system that consisted of two Exion AD pumps, an Exion AD multiplate autosampler set at 10 ± 2 ℃ and an Exion AC column oven set at 40 ± 2 ℃ (Sciex, Canada). Separation was performed using an Acquity HSS TS3 (1.8 μm, 2.1 mm I.D. × 100 mm) column (Waters). The mass spectrometer was a Sciex QTRAP 6500 + with a TurboIonSpray probe for electrospray ionization. Homogenization of brain samples was performed using a Precellys Evolution tissue homogenizer together with 2-mL lysing tubes containing ceramic beads for soft tissues (Bertin Technologies, France). Disposable 2-mL polypropylene Safe-Lock tubes (Eppendorf) were used for protein precipitation and preparation of standard solutions. Final sample preparations were performed in 2-mL 96-well plates from Eppendorf. AcroPrep 96 multiwell filter plates with a 350-µL reservoir and a 30-kDa Omega membrane (Pall Corporation, USA) were used for the ultrafiltration (UF) of the sample extracts.

#### Preparation of blood samples for LC–MS/MS

A 100-µL volume of blood was transferred to a disposable 2-mL tube and sequentially mixed with 50 µL of an internal standard (IS) solution containing 10 ng/mL each of quetiapine-D_8_ and norquetiapine-D_8_, 50 µL of MeOH and 300 µL of MeCN. After a standing time of 10 min, the mixture was centrifuged at 10,000×*g* for 5 min. A 300-µL volume of clear supernatant was transferred to a UF filter well. The filter unit was centrifuged at 2000×*g* for 5 min, and 250 µL of the filtrate was transferred to a glass-lined plate. The solvent was evaporated under a stream of nitrogen at 40 °C, and the residue was redissolved in 100 µL of 15% MeOH acidified with 0.1% FA.

#### Preparation of brain samples for LC–MS/MS

Each tissue sample (typically 50–150 mg for midbrain and 10–60 mg for PFC) was transferred to a lysing tube. A ninefold mass of cold water (approximately 4 °C) was added. The tissue was homogenized using the instrument's standard soft tissue program (speed 5800 rpm, cycle 2 × 15 s, pause 30 s). A 100-µL volume of the homogenate was transferred to a 2-mL tube and treated according to the procedure for blood samples.

#### Calibration of LC–MS/MS method

Calibrants based on blank rat blood and brain tissue were used to construct 6-point calibration curves. The samples were treated according to the above procedures, except that 50 µL of MeOH was replaced by 50 µL of mixed standard solutions of the drug substances prepared in MeOH. Calibrants were prepared at concentrations of 0.1, 1, 10, 100, 200, and 300 ng/mL in the blood and brain homogenate. The calibration curves were created by weighted (1/*x*) regression analysis of the IS-normalized peak areas (analyte area/IS area) using the quantifier product ions and their corresponding IS product ions. Because no labeled analog of O-dealkylquetiapine was commercially available, quetiapine-D_8_ was used for that component.

#### LC–MS/MS conditions

A 5 μL volume of the sample extract was injected into the column running 80% mobile phase A (0.1% FA in water) and 20% mobile phase B (0.1% FA in MeCN). The mobile phase was changed through a linear gradient to 65% A (35% B) over 5 min and then to 100% B over the next 0.5 min. Seven minutes after injection, the gradient was returned to 80% A (20% B) over 0.1 min, and the column was equilibrated for 1.9 min before the next injection, resulting in a total runtime of 9 min. The column flow rate was 400 μL/min, and the column temperature was maintained at 40 ± 2 °C. The eluent was diverted to waste at 0‒3.0 and 6.5‒9 min after injection using a post-column switch. The conditions used for multiple reaction monitoring (MRM) are listed in Table 1. The probe temperature (TEM) was set to 500 °C. The pressures of the curtain gas (CUR), ion source gas 1 (GS1), ion source gas 2 (GS2), and collision gas (CAD) were set at 20, 60, 60, and 9 psi, respectively. The mass spectrometer was operated in positive ion mode at unit mass resolution. The ion spray voltage was set at 2.5 kV. Nitrogen was used as the CAD gas. All analyte peaks were baseline separated to avoid interferences from in-source fragmentation of quetiapine and O-dealkylquetiapine. The retention times were 3.97 min for norquetiapine, 4.30 min for O-dealkylquetiapine, and 4.54 min for quetiapine.

**Table 1﻿:** Mass spectrometry conditions for the analysis of quetiapine and its metabolites

Substance	MRM transitions		DP (V)	EP	CE (eV)	CXP
	Q1 (*m/z*)	Q3 (*m/z*)		(V)		(V)
Quetiapine	384	**279**/247/221	90	8	35/53/51	23
Quetiapine-D_8_	392	**286**	90	8	35	23
Norquetiapine	296	221/210/**183**	30	6	39/40/53	21
Norquetiapine-D_8_	304	**183**	30	6	53	21
O-dealkylquetiapine	340	253/221/**210**	80	8	48/30/46	22

*DP* Declustering potential, *EP* Entrance potential, *CE* Collision energy, *CXP* Collision cell exit potential

The bold Q3 ions were used for quantification; the other ions were used for qualification

#### Method performance and quality control of LC–MS/MS analysis

The limits of detection (LODs) were determined from blank matrix samples (*n* = 10) spiked prior to extraction to concentrations resulting in an estimated signal-to-noise (S/N) ratio of approximately 3. The LOD was calculated as 2 × t_0.95_ × SD(C_S/N=3_) (t_0.95_ = 1.645). For blood the LODs were 0.05 ng/mL for quetiapine and O-dealkylquetiapine and 0.1 ng/mL for norquetiapine. For brain tissue the LODs were 0.5 ng/g (quetiapine), 0.5 ng/g (O-dealkylquetiapine), and 1 ng/g (norquetiapine). Accuracy and precision studies were performed using blank blood and brain homogenates spiked at different concentration levels. Duplicate analyses were performed on 6 different days. At concentrations of at least 1 ng/mL in blood and 10 ng/g in brain tissue, the relative intra-laboratory reproducibility standard deviation (RSD_R,intra-lab_) values were less than 10%, and the numerical biases were less than 5%. The lower limits of quantification (LLOQs) were determined from the same studies. The acceptance criteria for the LLOQs were a maximum RSD_R,intra-lab_ of 20% and a bias within ± 20%. This criterion was fulfilled for all analytes at a spiked concentration of 0.1 ng/mL in blood and 1 ng/g in brain tissue. The highest calibrant defined the upper limit of quantification (ULOQ). When the ULOQ was exceeded, blood samples were reanalyzed after dilution with blank blood. Brain homogenates were diluted with water, and a similar diluted matrix was used for calibration. The mean extraction recoveries were for all analytes greater than 95% at concentration levels of 1 ng/mL and 100 ng/mL in blood and 10 ng/g and 1000 ng/g in brain tissue (*n* = 10 for each).

Blood and brain samples were analyzed with at least two single determinations performed in different batches. Two matrix-matched quality control samples at analyte concentrations of 1 ng/mL and 100 ng/mL in blood and brain homogenates were included in each analytical sequence.

### ﻿Reporting of data and data analysis

After Pareto scaling, principal component analysis (PCA) was applied to each untargeted metabolomics dataset for initial data evaluation using SIMCA^®^ version 16.0.1 (Sartorius Stedim Data Analytics AB, Göttingen, Germany). Prior to PCA, we removed features related to quetiapine, methadone, and their metabolites.

A two-sample *t*-test with equal or unequal variance based on an *F*-test was used to evaluate significant differences between two sample groups. Histograms and QQ plots were used to assess the distribution of data. A *p*-value < 0.05 was considered significant.

Untargeted data are presented as boxplots showing the minimum, first quartile, median, third quartile, and maximum. Quantitative data are presented as means with error bars representing standard deviation (SD) in graphs.

## Results

### Profiling of quetiapine, methadone, and their metabolites

Brain and blood samples were obtained from 9, 8–9, 9–11, and 8–11 animals in the QTP, MTD, QTP + MTD, and control groups, respectively. Methadone and quetiapine were detected in both blood and brain within their respective treatment groups. For those rats administered quetiapine, we additionally found norquetiapine, O-dealkylquetiapine, 7-hydroxyquetiapine, and quetiapine sulfoxide in all three matrices (midbrain, PFC, and blood) with our untargeted approach. Quetiapine acid and EDDP, a metabolite of methadone, were found in blood and midbrain but not in PFC (Figure S1 and S2). Metabolites of quetiapine and methadone were tentatively annotated based on *m/z* and fragmentation patterns (Figure S3). We did not detect EMDP, likely due to concentrations below our detection limit.

**Fig. 1:**
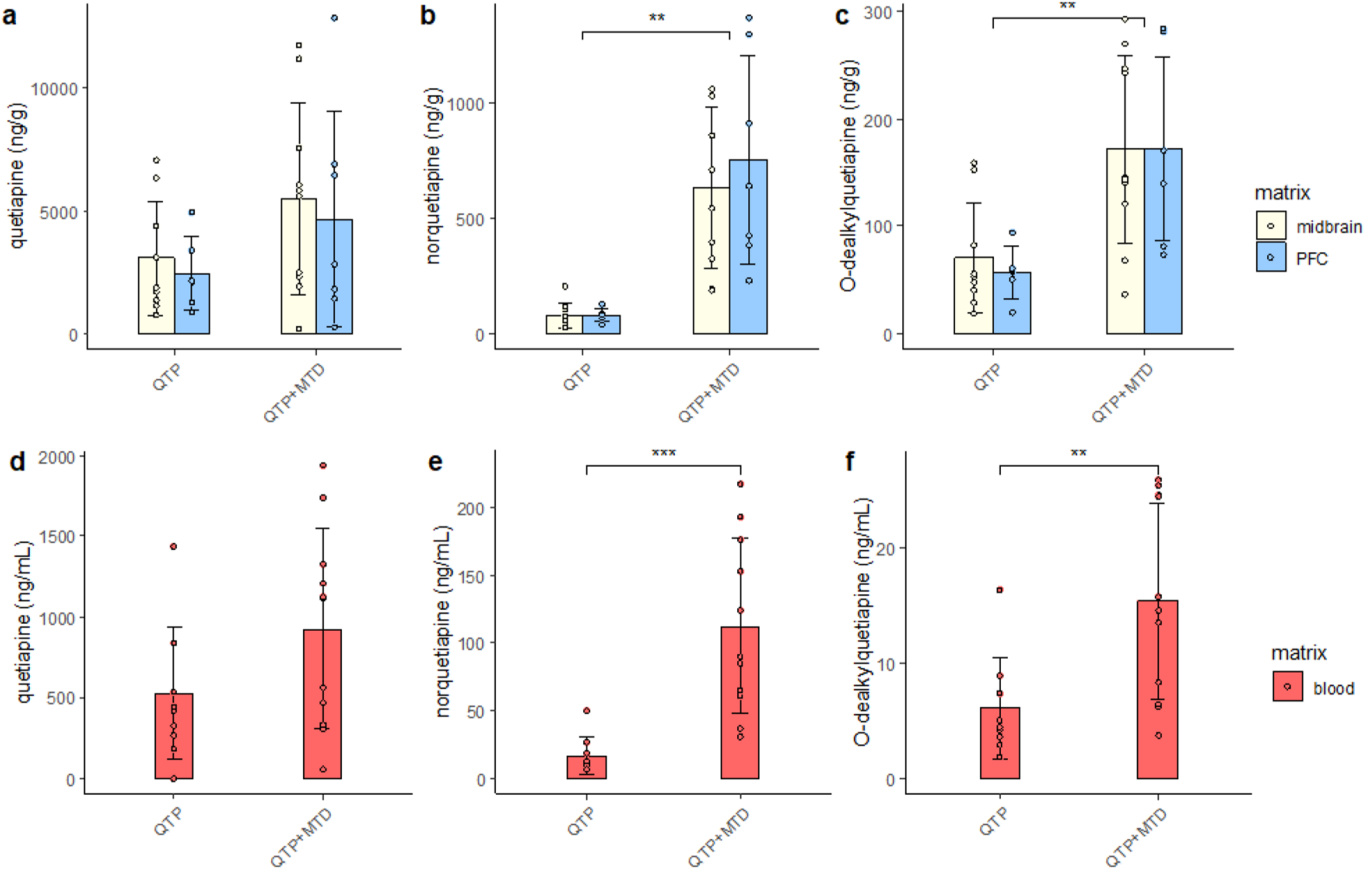
Concentrations of quetiapine, norquetiapine and O-dealkylquetiapine in brain (**a**, **b**, **c**) and blood (**d**, **e**, **f**) of rats receiving quetiapine (QTP, *n* = 9) or quetiapine + methadone (QTP + MTD, *n* = 11). Bars represent mean concentrations with standard deviations. Individual values are made in circles. Midbrain (yellow), PFC (blue), and blood (red). PFC = prefrontal cortex. Significant differences are marked with asterisks (**p < 0.01, ***p < 0.001)

Methadone and EDDP concentrations were decreased in both PFC and midbrain in the treatment group receiving both quetiapine and methadone (QTP + MTD) compared to MTD alone (*p* < 0.05, Figure S1). The QTP + MTD group showed a general up-regulation of quetiapine metabolites in all three matrices compared to the QTP group (Figure S2). We further verified this up-regulation by quantifying quetiapine, norquetiapine, and O-dealkylquetiapine within the QTP and QTP + MTD treatment groups (Fig. 1). Increased concentrations of norquetiapine and O-dealkylquetiapine were significant in both blood and brain (*p* < 0.01) of rats receiving quetiapine in combination with methadone. Quetiapine was not significantly changed between the two groups but showed an increasing trend in the QTP + MTD group.

**Fig. 2:**
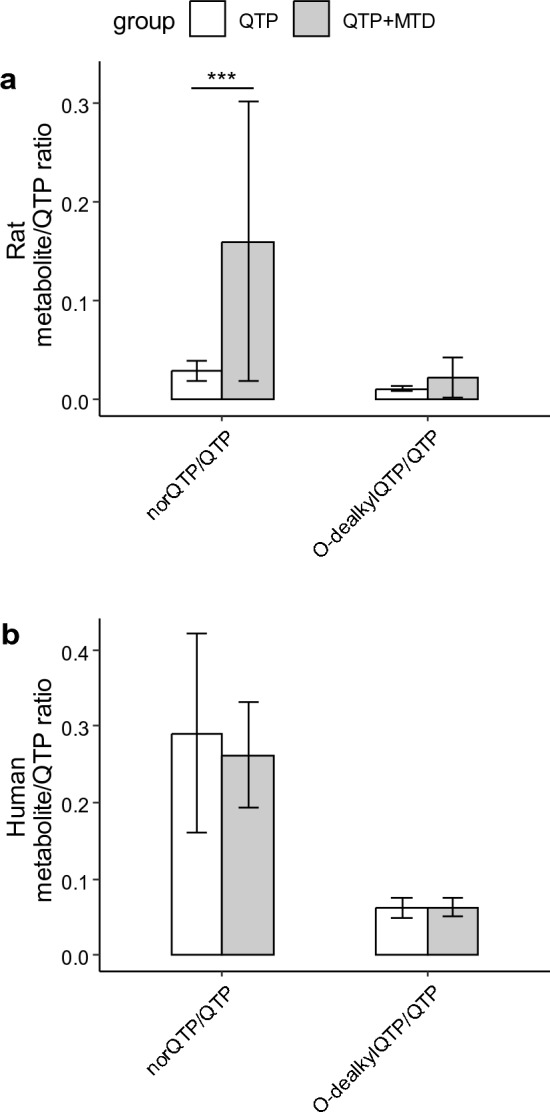
Quantification ratios of norquetiapine and O-dealkylquetiapine vs. quetiapine in the blood of rats (**a**) and blood from a cohort of DUID humans tested positive for quetiapine (QTP) or quetiapine + methadone (QTP + MTD) (**b**). Human samples were filtered to only include ratios between norquetiapine and quetiapine below 0.5 (n_QTP_ = 17, n_QTP+MTD_ = 14) and O-dealkylquetiapine and quetiapine below 0.08 (n_QTP_ = 11, n_QTP+MTD_ = 14) similar to the rats. Bars represent mean ratios with standard deviations. norQTP = norquetiapine, O-dealkylQTP = O-dealkylquetiapine. Significant differences are marked with asterisks (***p < 0.001). Data were log-transformed before statistical significance testing

A significant difference in the ratio of norquetiapine vs. quetiapine in blood and brain from rats was evident between the two treatment groups QTP + MTD and QTP, whereas no differences in the ratio of O-dealkylquetiapine vs. quetiapine were found (Fig. 2a and Figure S4). In human blood samples, there was no significant difference in the norquetiapine: quetiapine or O-dealkylquetiapine: quetiapine concentration ratio, respectively, between people having both quetiapine and methadone in their bloodstream vs. those only with quetiapine (data not shown). As this was not a controlled human study, we tried to filter the data only to include subjects having ratios between norquetiapine vs. quetiapine below 0.5 and O-dealkylquetiapine vs. quetiapine below 0.08, similar to the rats. However, no significant changes in the quantification ratios were observed between the two groups of subjects (Fig. 2b).

### Untargeted profiling of endogenous metabolites affected by quetiapine and methadone

**Fig. 3:**
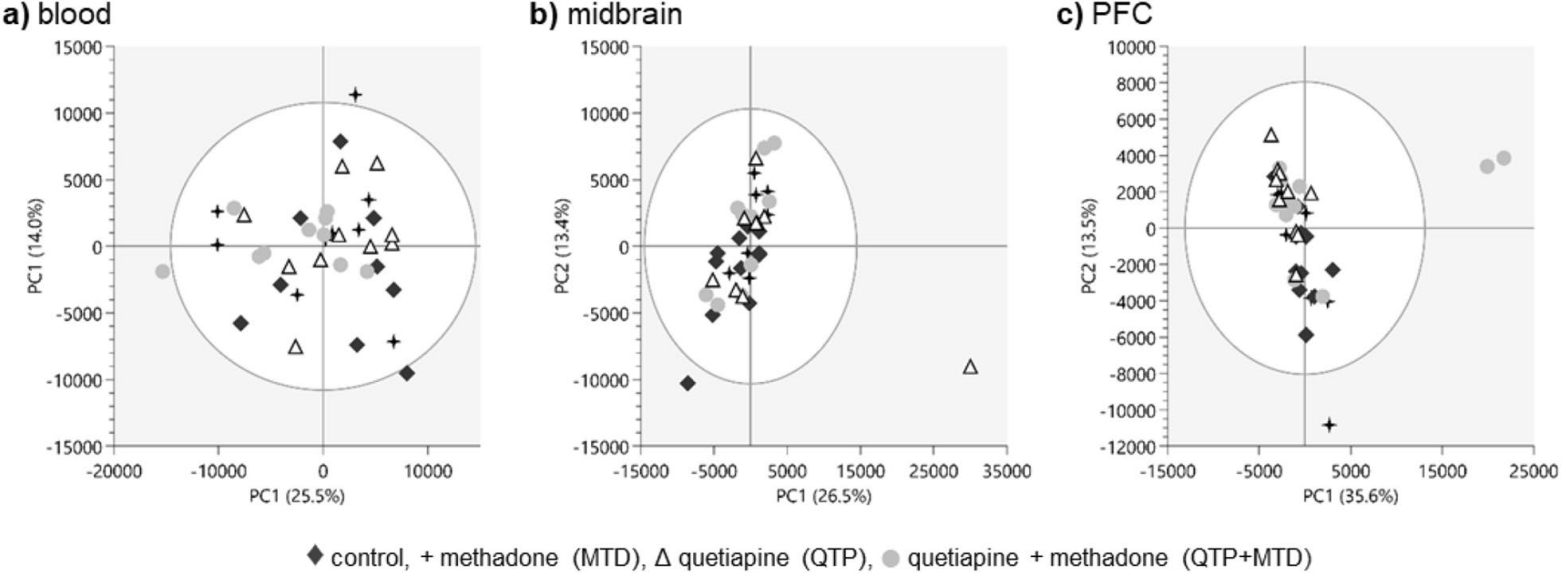
PCA scores plots of all features (except the drugs and their associated metabolites) following data filtering, normalization, and scaling in blood (**a**), midbrain (**b**), and PFC (**c**) in positive ESI mode. Treatment groups are given as control (filled diamond), methadone (MTD filled star), quetiapine (QTP unfilled triangle), and quetiapine + methadone (QTP + MTD (filled circle))

Based on the untargeted UPLC-HR-QTOF-MS analysis, we did a PCA (excluding the drugs and their associated metabolites) to look for endogenous changes in blood, midbrain, and PFC following acute intoxication with co-administration of quetiapine and methadone. Following data filtering, normalization, and scaling, the PCA scores plots did not show any clustering to the four assigned treatment groups in any of the PCA components. This was true for all three matrices in both positive (Fig. 3) and negative ionization modes (Figure S5). Not even the control group seemed to cluster separately. Despite this, we looked further into metabolites showing a significant alteration between rats receiving QTP and MTD versus QTP + MTD using two-sample t-tests.

We annotated 296, 225, and 277 endogenous metabolites in blood, midbrain, and PFC, respectively, subject to further statistical analysis. Several of these metabolites showed significant differences between one or more treatment groups compared to control. Significant differences between rats receiving QTP + MTD vs single administrations of either QTP or MTD were, however, limited to N-methylglutamic acid, glutaric acid, and p-hydroxyphenyllactic acid in PFC, and corticosterone in midbrain (*p* < 0.05) – all verified by standard comparisons (*m*/*z*, fragments, and retention time).

**Fig. 4:**
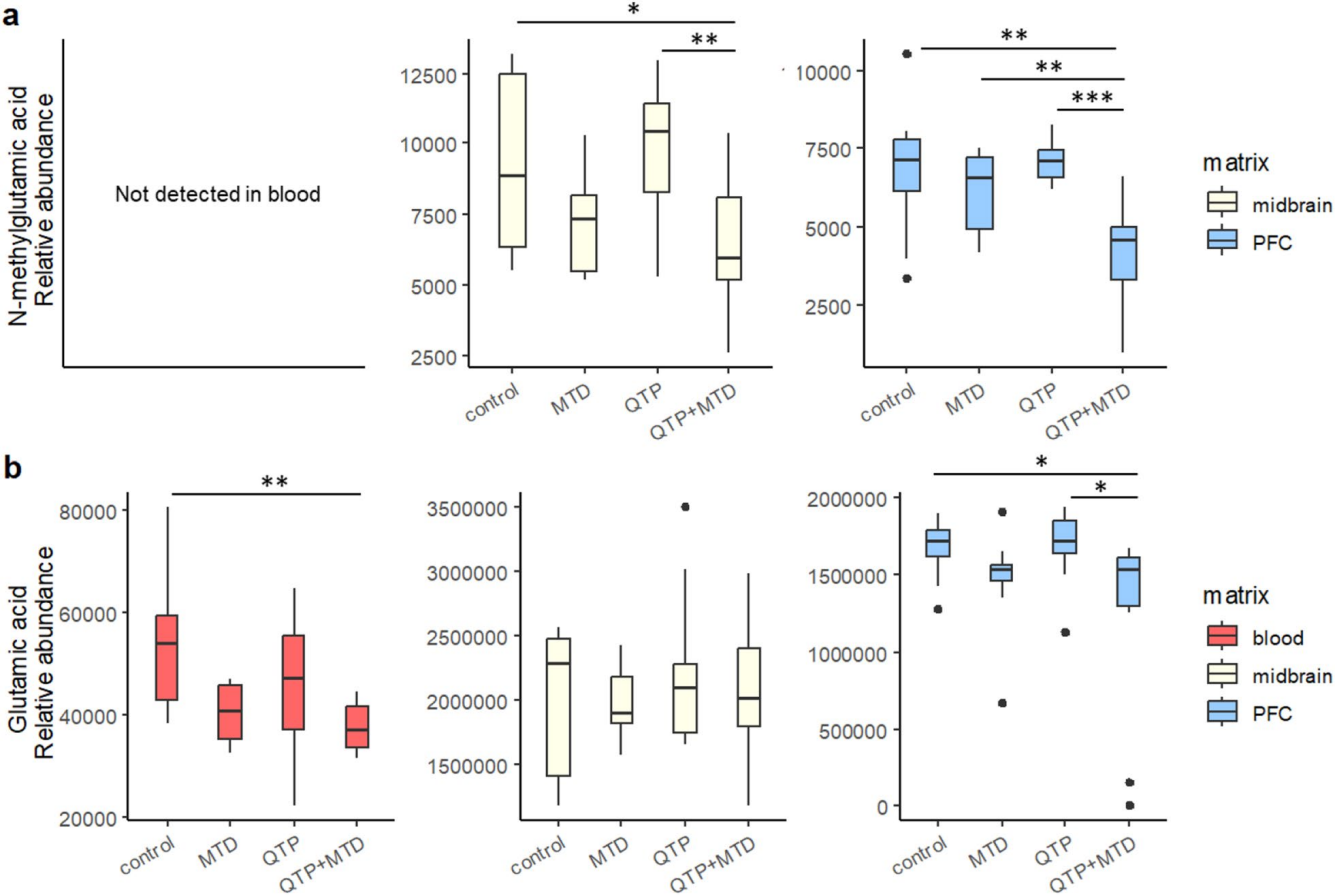
Boxplots of the relative levels of N-methylglutamic acid (**a**) and glutamic acid (**b**) in blood (red), midbrain (yellow), and PFC (blue) of rats. Depicted is the relative abundance (normalized peak area) of each analyte for each treatment group: control, methadone (MTD), quetiapine (QTP), and quetiapine + methadone (QTP + MTD). N-methylglutamic acid was not detected in blood. Significant differences to QTP + MTD are marked with asterisks (*p < 0.05, **p < 0.01, ***p < 0.001). Outliers are given as dots (colour figure online)

N-methylglutamic acid, a chemical derivative of glutamic acid, was significantly decreased in PFC of rats receiving QTP + MTD compared to the other three treatment groups (*p* < 0.01, Fig. 4a). A significant effect was also seen in the midbrain but not compared to MTD alone. Glutamic acid was significantly decreased in PFC in the QTP + MTD group compared to control and QTP, but not in MTD (Fig. 4b). Glutamic acid was additionally decreased in blood compared to the control.

**Fig. 5:**
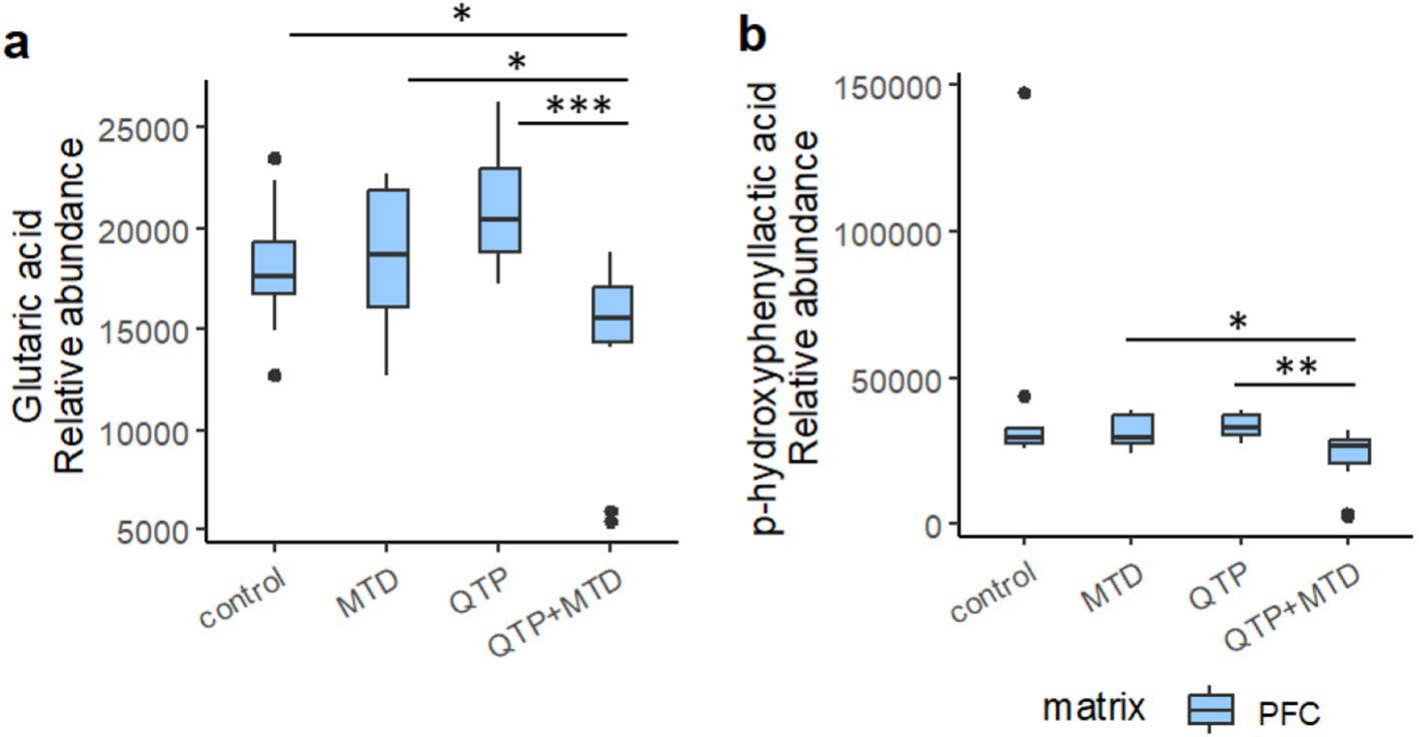
Boxplots of the relative levels of glutaric acid (**a**) and p-hydroxyphenyllactic acid (**b**) in PFC of rats. Depicted is the relative abundance (normalized peak area) of each analyte for each treatment group: control, methadone (MTD), quetiapine (QTP), and quetiapine + methadone (QTP + MTD). Significant differences are marked with asterisks (*p < 0.05, **p < 0.01, ***p < 0.001). Outliers are given as dots

Glutaric acid and p-hydroxyphenyllactic acid were significantly decreased in PFC of rats receiving QTP + MTD compared to single administrations of QTP or MTD (Fig. 5a and b). Glutaric acid was also significantly decreased in the QTP + MTD group compared to control. No differences were observed in the midbrain, and none of the compounds were detected in the blood.

**Fig. 6:**
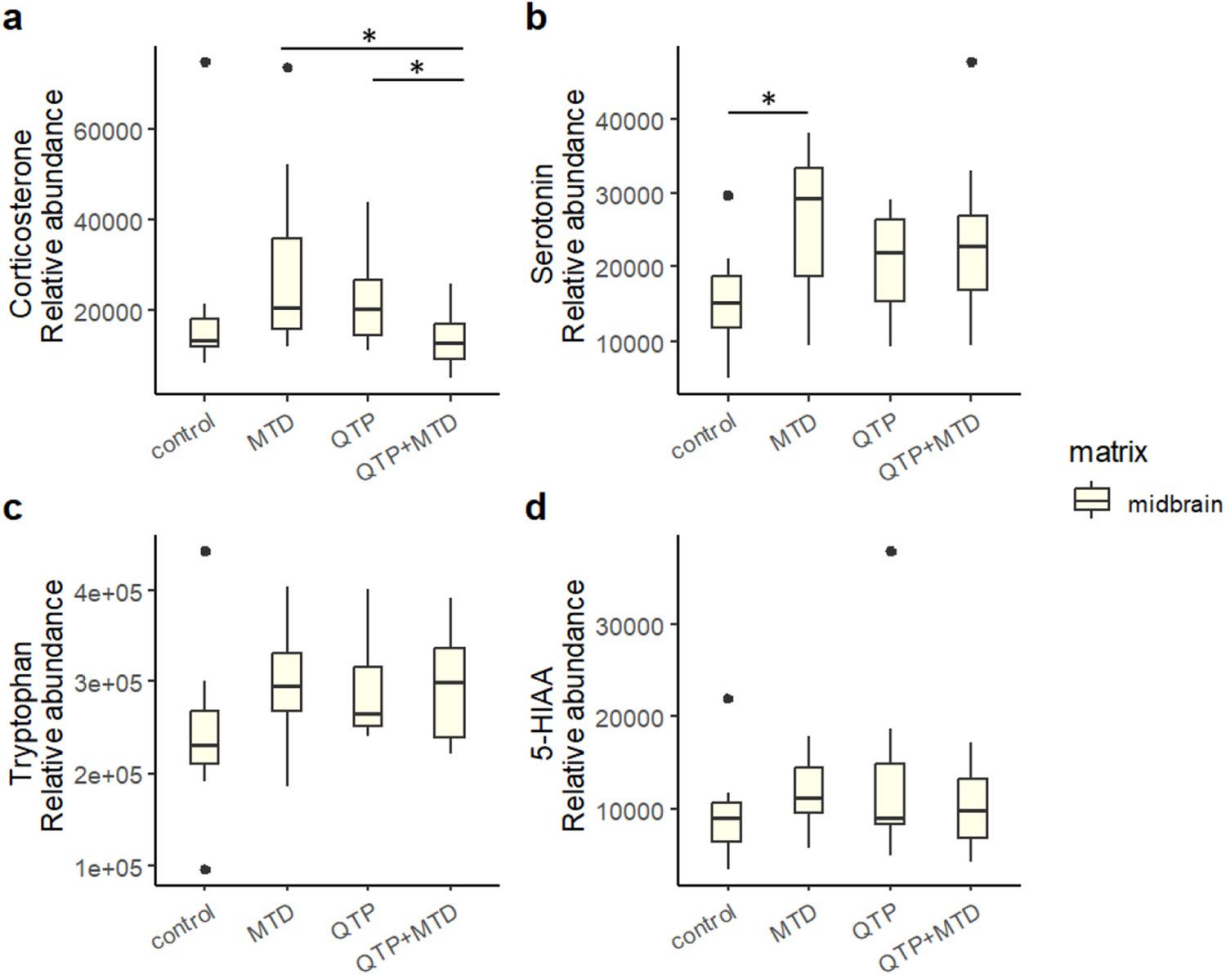
Boxplots of the relative levels of corticosterone (**a**), serotonin (**b**), tryptophan (**c**), and 5-hydroxy-indole-3-acetic acid (5-HIAA) (**d**) in the midbrain of rats. Depicted is the relative abundance (normalized peak area) of each analyte for each treatment group: control, methadone (MTD), quetiapine (QTP), and quetiapine + methadone (QTP + MTD). Significant differences are marked with asterisks (*p < 0.05). Outliers are given as dots

Corticosterone was found in the blood and midbrain of rats but not detected in PFC. It was significantly decreased in the midbrain of rats receiving QTP + MTD compared to QTP or MTD (Fig. 6a).

Even though not significantly different in the QTP + MTD group, it was noticeable that methadone treatment was associated with increased serotonin levels in the midbrain (Fig. 6b). Serotonin was not detected in PFC. The levels of tryptophan (precursor to serotonin) and 5-hydroxy-indole-3-acetic acid (5-HIAA, degradation product of serotonin) did not show significant down- or up-regulation in the presence of methadone that could explain this increase (Fig. 6c and d).

Additional metabolites in blood showing significant differences between rats receiving QTP + MTD vs single administrations of both QTP and MTD are listed in Table 2. In all cases, the metabolites are slightly up-regulated in the QTP + MTD group compared to single administrations.

**Table 2:** Metabolites in blood that show significant differences between rats receiving quetiapine + methadone (QTP + MTD) versus single administrations of quetiapine (QTP) and methadone (MTD)

Metabolite	Fold change: (QTP + MTD)/QTP	Fold change: (QTP + MTD)/MTD
2-hydroxybutyric acid^a^	1.4*	1.5*
2-methylglutaric acid^a^	1.2*	1.4**
4-imidazoleacetic acid^a^	1.4**	1.3**
5-methyluridine^b^	1.4***	1.3**
LysoPE(20:1)^b^	1.7*	1.6*

^a^Verified by standard compounds (*m*/*z*, fragments and retention time)

^b^Verified by *m*/*z* and fragments

*p < 0.05

**p < 0.01

***p < 0.001

## Discussion

This study suggested pharmacokinetic interactions between methadone and quetiapine in terms of increased levels of quetiapine metabolites in rats' blood and brain and decreased methadone and EDDP concentrations in the brain when the drugs were combined. However, alterations in the endogenous metabolites in response to the mix of the drugs were limited. These results align with the previous study by Andersen et al. (2022) on the same rats, which found no additive effects on sedation and locomotor activity.

It is worth noting that all the identified quetiapine metabolites have previously been observed in humans and that rats have orthologous CYP proteins with functions similar to CYP3A4/5 and CYPD26 (Hammer et al. 2021; Zuber et al. 2002). However, variations in species-specific expression levels may cause differences in metabolic pathways and kinetics. We tried to verify our findings in human DUID cases but did not find a similar effect. However, the uncontrolled nature of the human samples, concerning, e.g., time of intake and blood sampling, ingested doses, and possible influence of other drugs, necessitates caution when interpreting this result. Therefore, to confirm whether the observed changes in quetiapine metabolism in the presence of methadone are similar in humans, a controlled study involving human subjects would be required.

It is known that methadone inhibits CYP2D6 in humans (Gelston et al. 2012), which could explain the up-regulation of some of the quetiapine metabolites if there is a similar inhibition in orthologous CYP proteins in rats. In humans, CYP3A4/5 metabolizes quetiapine into norquetiapine, O-dealkylquetiapine, and quetiapine sulfoxide, which CYP2D6 further metabolizes. Blocking CYP2D6 would lead to the accumulation of these three metabolites in tissues and blood, which seems to be the case. Although some previous studies indicate that methadone can inhibit CYP3A4 (Boulton et al. 2001; Iribarne et al. 1997), quetiapine itself was not significantly higher in the QTP + MTD group, indicating that CYP3A enzymes were still active.

In the presence of quetiapine, methadone and its metabolite EDDP were decreased in the rats' two brain sections. Boulton et al. 2002 have shown that quetiapine is a suitable substrate for the P-GP transporter and stimulates P-GP ATPase activity at low concentrations (Boulton et al. 2002). This stimulation could perhaps lead to increased transport of methadone and EDDP out of the brain into the bloodstream. Methadone itself has also been shown to be a substrate for P-GP in rats (Rodriguez et al. 2004). However, P-GP activity has been shown to be higher in rats compared to humans in certain cases (Al Feteisi et al. 2018; Verscheijden et al. 2021), suggesting that this effect may not be as pronounced in humans.

Furthermore, four endogenous metabolites were found to be decreased in the brain of the QTP + MTD group compared to both the QTP and MTD groups. One of these metabolites, N-methylglutamic acid, is a chemical derivative of glutamic acid, the brain's most abundant neurotransmitter. Demethylation of N-methylglutamic acid by methylglutamate dehydrogenase leads to the formation of glutamic acid. The observed decrease of N-methylglutamic acid could be an attempt to maintain sufficient neurotransmitter levels. This suggests that, in combination, methadone and quetiapine reduce glutamic acid stores, which the cells attempt to compensate for using glutamic acid derivatives. A decrease in glutamic acid levels was also observed in the PFC and blood in the QTP + MTD group compared to the control group (and QTP in PFC), but not in the MTD group. Previously, Greenwald et al. (2015) have demonstrated that high doses of methadone decrease glutamic acid levels in the anterior cingulate cortex, a part of the PFC, in heroin-dependent individuals during methadone maintenance treatment (Greenwald et al. 2015). Based on our findings, this effect is enhanced in the presence of quetiapine in rats.

Both glutaric acid and p-hydroxyphenyllactic acid (the L-form) are naturally produced in the body from the metabolism of lysine, tryptophan, and tyrosine. The decrease in these compounds observed in the QTP + MTD group may be attributed to other demands for these amino acids in the PFC, whereby they are not metabolized to these specific products.

Corticosterone is the main glucocorticoid in animals such as rodents, while cortisol is the primary active glucocorticoid in humans. Corticosterone is secreted in response to environmental challenges and has an important function in the regulation of energy and stress responses. A previous study has shown that a single 0.2 mg/kg dose of methadone effectively reduced the degradation of cortisol to 6b-hydroxycortisol indicated by a decreased urinary 6b-hydroxycortisol to cortisol ratio (Boulton et al. 2001). Our study observed a decrease in corticosterone in the midbrain when methadone and quetiapine were co-administered compared to single administrations. This decrease could be connected to the effect of quetiapine, which may cause methadone to be transported out of the brain by increasing the activity of the P-GP transporter and result in corticosterone levels similar to the control group.

In the midbrain, we found an increase in serotonin levels in the MTD group compared to the control group. This increase is likely due to an overall up-concentration in the brain cells. We looked for changes in the precursor tryptophan and the degradation product 5-HIAA. This was to evaluate whether there was an increased formation of serotonin or perhaps an inhibition of the monoamine oxidase (MAO) or aldehyde dehydrogenase (ALDH) that degrades serotonin. However, we found no significant differences between the treatment groups. Thus, we are currently unaware of the mechanism behind this accumulation. The QTP + MTD group did not exhibit the same significant increase in serotonin, which maybe also could be attributed to increased P-GP activity in the presence of quetiapine.

In contrast to the decreased metabolites observed in the brain, the metabolites of interest in the blood were all slightly increased. These compounds have diverse functions, suggesting that several processes, e.g., in the liver, may be affected. For example, the metabolite 2-hydroxybutyrate is known to appear at high concentrations in situations related to oxidative stress and detoxification demands (Gall et al. 2010; Zheng et al. 2013), which also seems plausible in this context. The methylated nucleoside 5-methyluridine could indicate increased post-transcriptional modifications, potentially leading to changes in protein synthesis. However, the relationship of these compounds to the intake of methadone and quetiapine is otherwise unknown.

We found no additive effects of quetiapine and methadone on metabolism, indicating increased toxicity. In contrast, quetiapine attenuated some changes induced by methadone, such as increased serotonin and corticosterone. This agrees with the previous study by Andersen et al. (2022), in which no additive effects on sedation or locomotor activity were observed, as well as a recent study in which quetiapine attenuated conditioned place preference and decreased locomotor activity induced by morphine (Khezri et al. 2022). In the study by Khezri et al., the attenuating effect was associated with reduced phosphorylation of extracellular signal-regulated kinase in the hippocampus, whereas it was not investigated if pharmacokinetic interactions could play a role as in our study. Interestingly, it has been shown that 250 mg/kg quetiapine given orally to rats resulted in increased respiratory depression induced by oxycodone (Xu et al. 2021), and that this was most likely due to increased oxycodone concentrations. In the study by Xu et al. (2021), the peak concentrations of quetiapine were comparable to those measured in the present study. Thus, the interactions between quetiapine and different opioids may vary and should not be generalized.

The main limitation of this study lies in the uncertainty of extrapolating the results to humans. Although rats have orthologous CYP proteins, the pathways and kinetics may differ between species. We could not verify the observed changes in quetiapine metabolism in human DUID cases, although this result should be interpreted cautiously. Extrapolation of the doses from rats to humans is also uncertain (Andersen et al. 2022). The duration from the first dose until the rats were sacrificed was furthermore relatively short (~ 1.5 h), which could influence our results compared to other studies. On another note, we chose to use two specific brain regions because changes in metabolism and neurotransmitters can differ between brain regions in response to stimuli and thus be canceled out if whole-brain homogenized samples are used. We chose to use the PFC and the midbrain due to evidence demonstrating that the vigilance is determined by regulation of PFC activation by the arousal system in the midbrain in a coordinated fashion (Wu et al. 2023). Other brain areas might have been of interest for the study, and the evaluation of only two brain regions remains as a limitation of the present investigation. Furthermore, both the PFC and the midbrain comprise many smaller brain regions with distinct functions in which the drug effect might have been canceled out by the absence of more detailed dissection. Finally, the identified endogenous metabolites in this study would require confirmation through targeted studies to validate the findings.

## Conclusion

In conclusion, this study in rats suggests pharmacokinetic interactions between quetiapine and methadone, resulting in increased blood and brain concentrations of quetiapine metabolites and decreased brain concentrations of methadone and EDDP. Furthermore, quetiapine seemed to attenuate methadone-induced increases in brain serotonin and corticosterone levels, suggesting that quetiapine may counteract some potential adverse effects of methadone. The differences between rat and human metabolic pathways are important considerations for future research.

### Supplementary Information

Below is the link to the electronic supplementary material.

Supplementary file1 (XLSX 632 KB)

Supplementary file2 (DOCX 515 KB)

## Data Availability

Raw *mzML*.-files are available upon request to the corresponding author. The annotated metabolites with corresponding data are provided in the supplementary spreadsheet.
