# Supplementary material for: Combined in vivo metabolic effects of quetiapine and methadone in brain and blood of rats
Source: Arch Toxicol. 2023 Oct 23;98(1):289–301. doi: 10.1007/s00204-023-03620-2 (PMC10761411; doi:10.1007/s00204-023-03620-2)
Supplement: Supplementary file 2 — Supplementary file2 (DOCX 515 KB) [file 204_2023_3620_MOESM2_ESM.docx]

Supplementary Information (SI)

# Combined *in vivo* metabolic effects of Quetiapine and methadone in brain and blood of rats

Laura Smedegaard Heisel^1^, Freja Drost Andersen^1^, Sâmia Joca^2^, Lambert Kristiansen Sørensen^1^, Ulf Simonsen^2^, Jørgen Bo Hasselstrøm^1^, Charlotte Uggerhøj Andersen^1,2,3^, Kirstine Lykke Nielsen^1^*

1. Department of Forensic Medicine, Aarhus University, Palle Juul-Jensens Boulevard 99, DK-8200 Aarhus N
2. Department of Biomedicine, Aarhus University, Høegh-Guldbergs Gade 10, DK-8000 Aarhus C
3. Department of Clinical Pharmacology, Aarhus University Hospital, Palle Juul-Jensens Boulevard 99, DK-8200 Aarhus N

* Corresponding Author: [klyn@forens.au.dk](mailto:klyn@forens.au.dk)


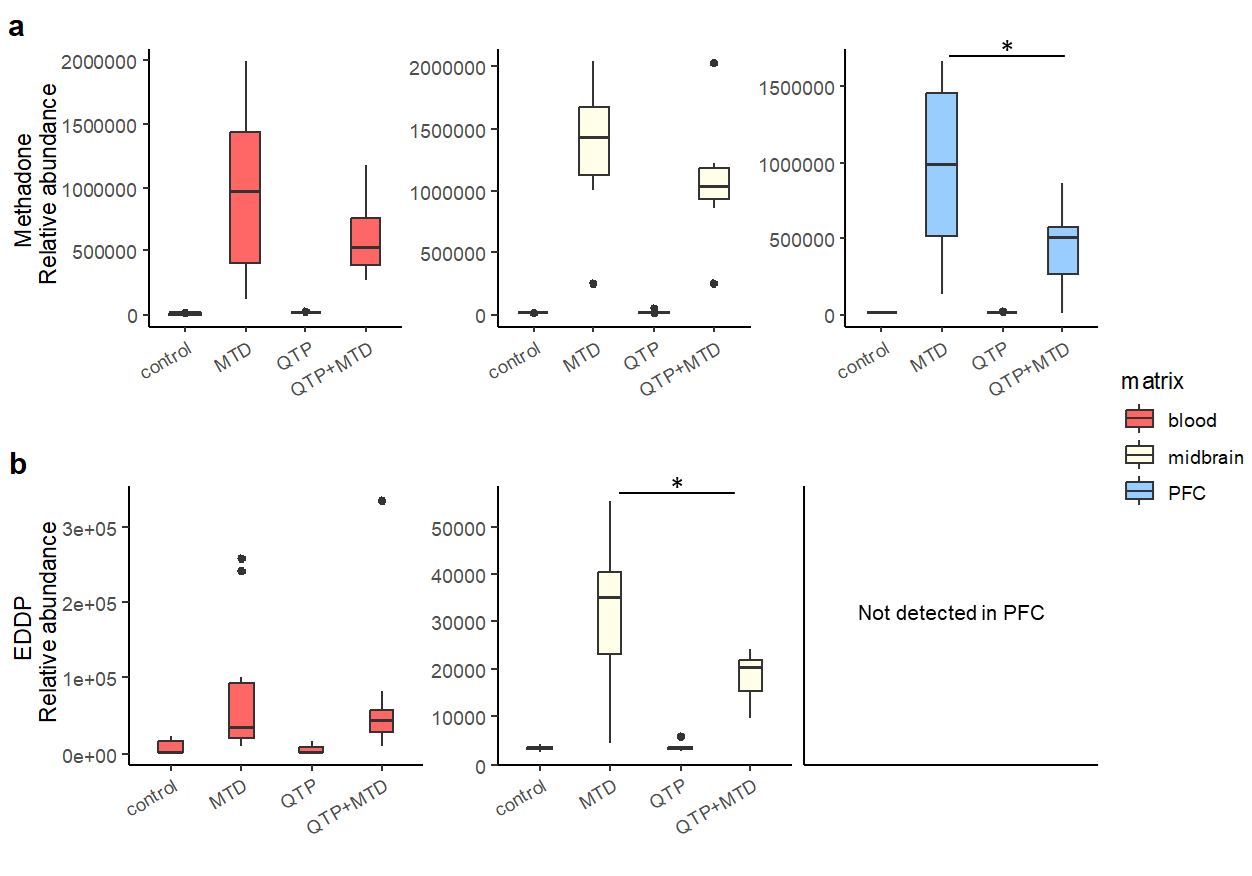


**Fig. S1** Boxplots of the relative levels of methadone (a) and its metabolite EDDP (b) in the blood (red), midbrain (yellow), and PFC (blue). Depicted is the relative abundance (normalized peak area) of each analyte for each treatment group: control, methadone (MTD), quetiapine (QTP), and quetiapine + methadone (QTP+MTD). EDDP was not detected (ND) in PFC. Significant differences between the MTD and QTP+MTD are marked with asterisks (**p* < 0.05). Methadone and EDDP were not detected in the control or QTP groups, but baseline levels from XCMS are depicted. Outliers are given as dots.


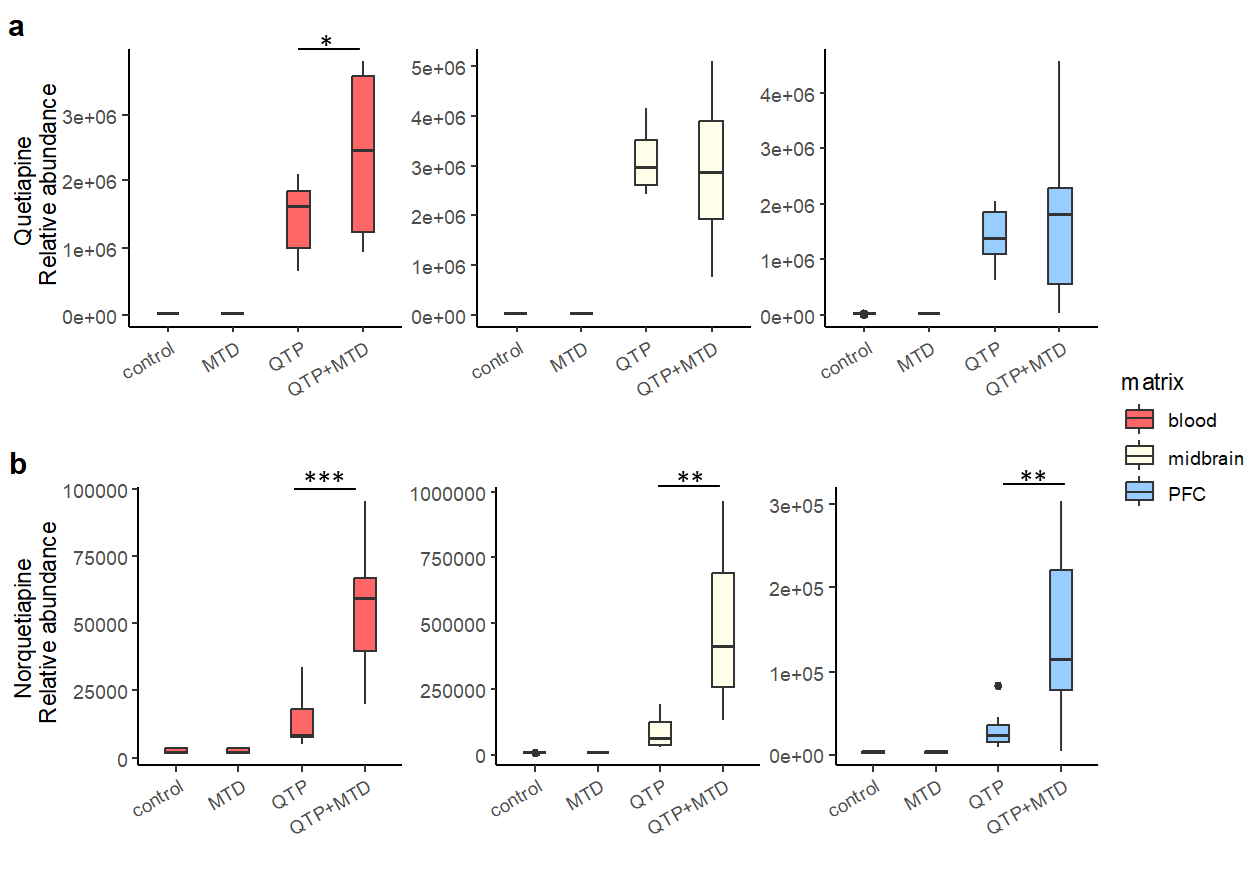


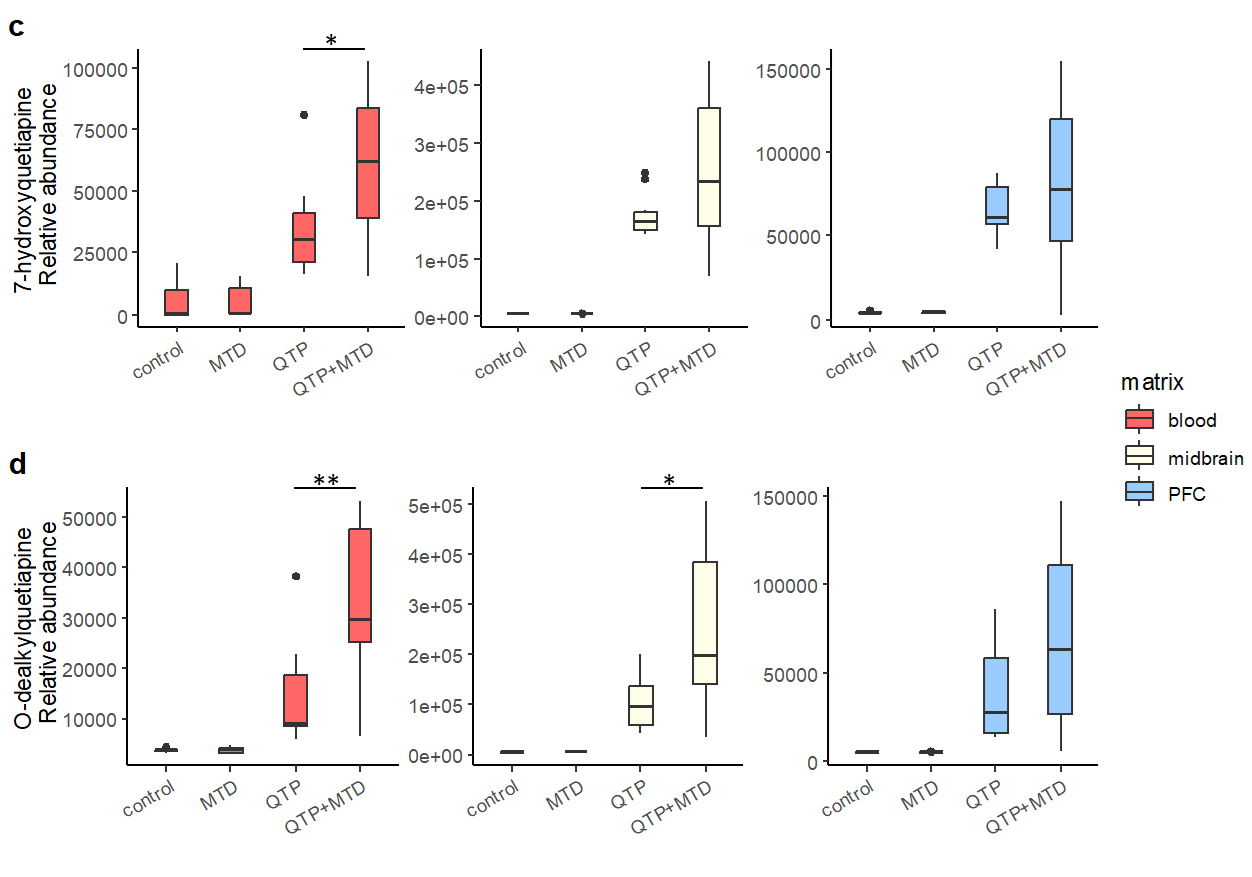


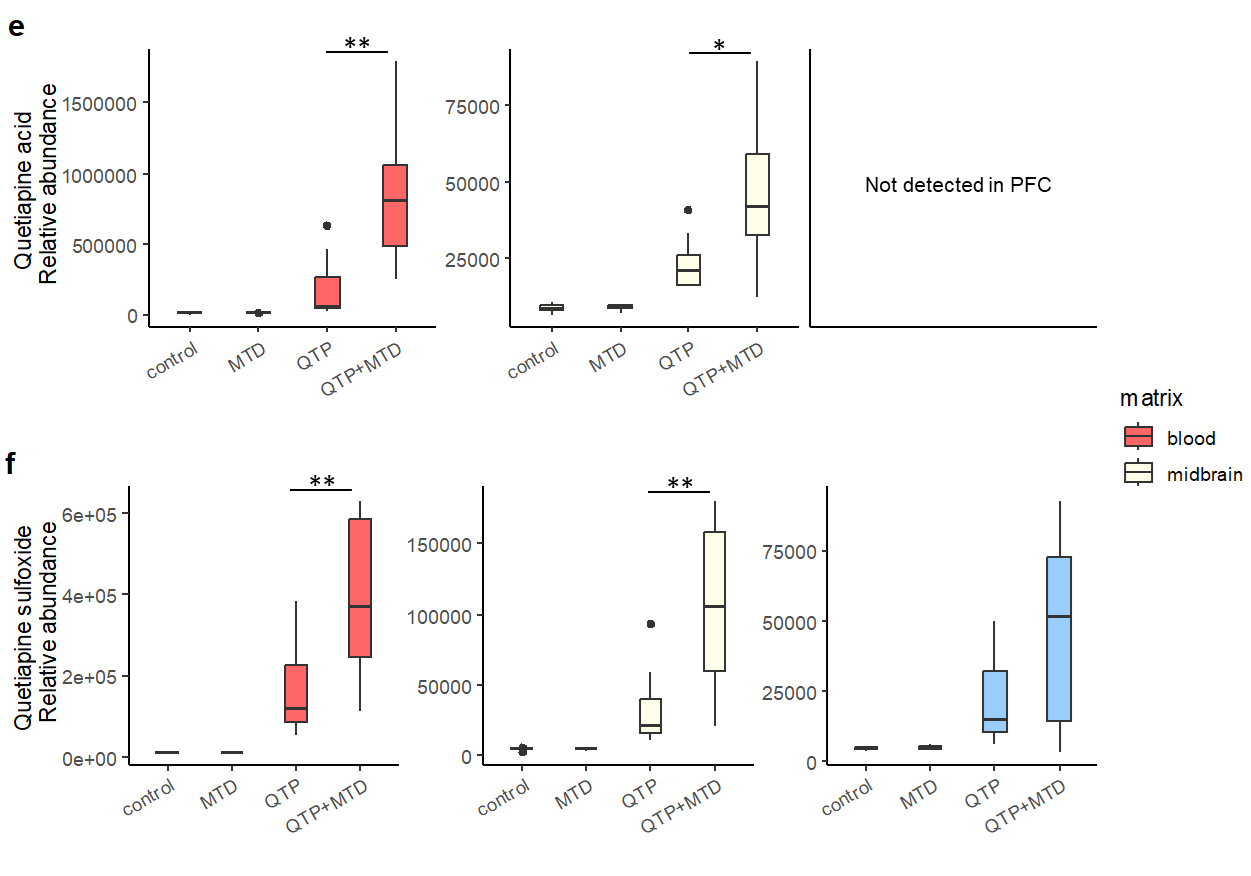


**Fig. S2** Boxplots of the relative levels of quetiapine (a) and all its detected metabolites; norquetiapine (b), 7-hydroxyquetiapine (c), O-dealkylquetiapine (d), quetiapine acid (e) and quetiapine sulfoxide (f) in blood (red), midbrain (yellow) and PFC (blue). Depicted is the relative abundance (normalized peak area) of each analyte for each treatment group: control, methadone (MTD), quetiapine (QTP) and quetiapine + methadone (QTP+MTD). Quetiapine acid was not found in PFC. Significant differences between QTP and QTP+MTD are marked with asterisks (****p* < 0.001, ***p* < 0.01, **p* < 0.05). Quetiapine and metabolites were not detected in the control or MTD groups, but baseline levels from XCMS are depicted. Outliers are given as dots.


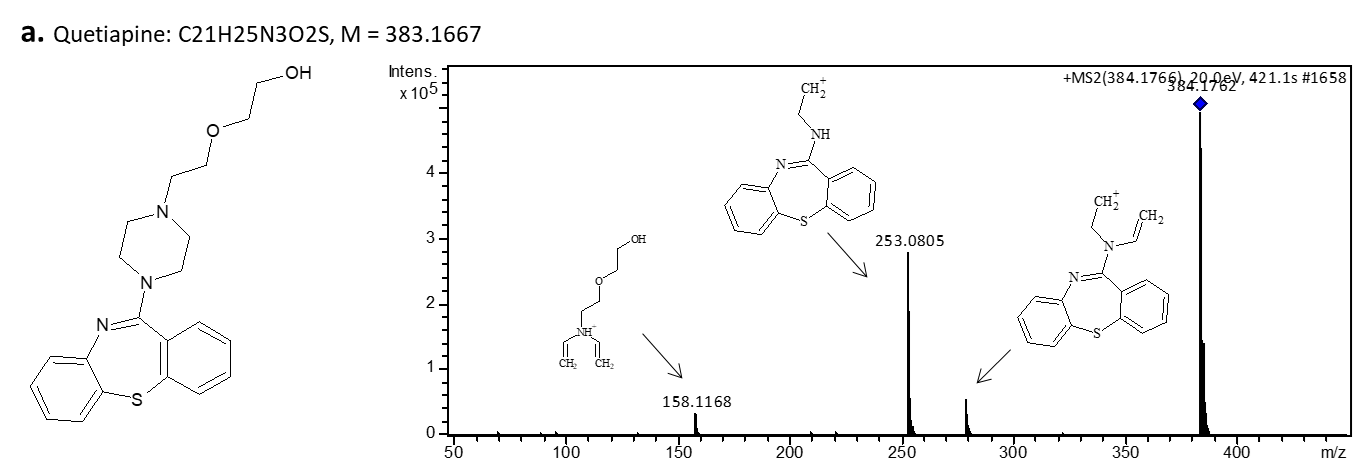


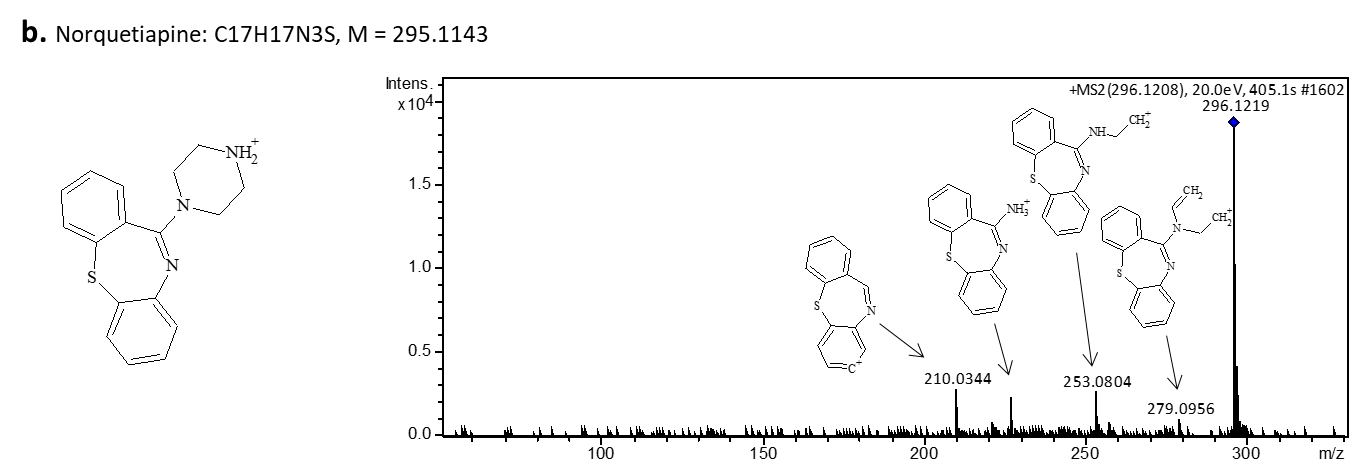


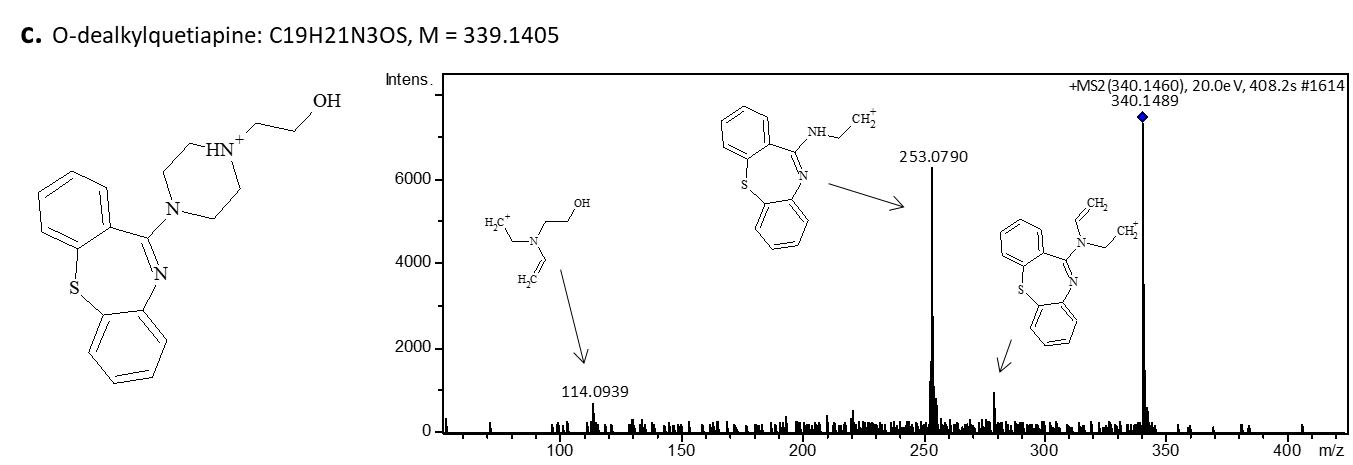


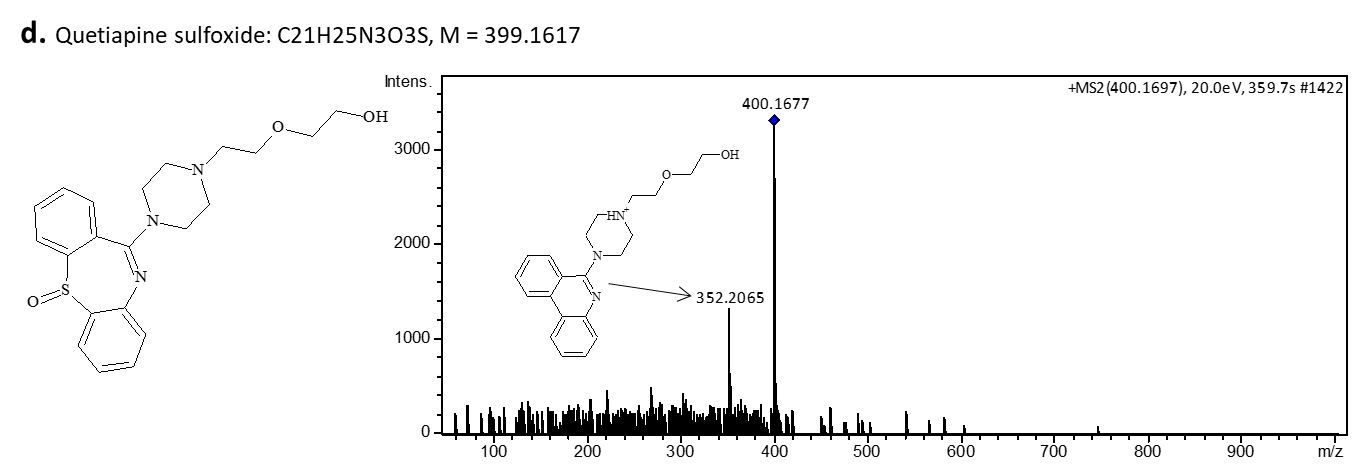


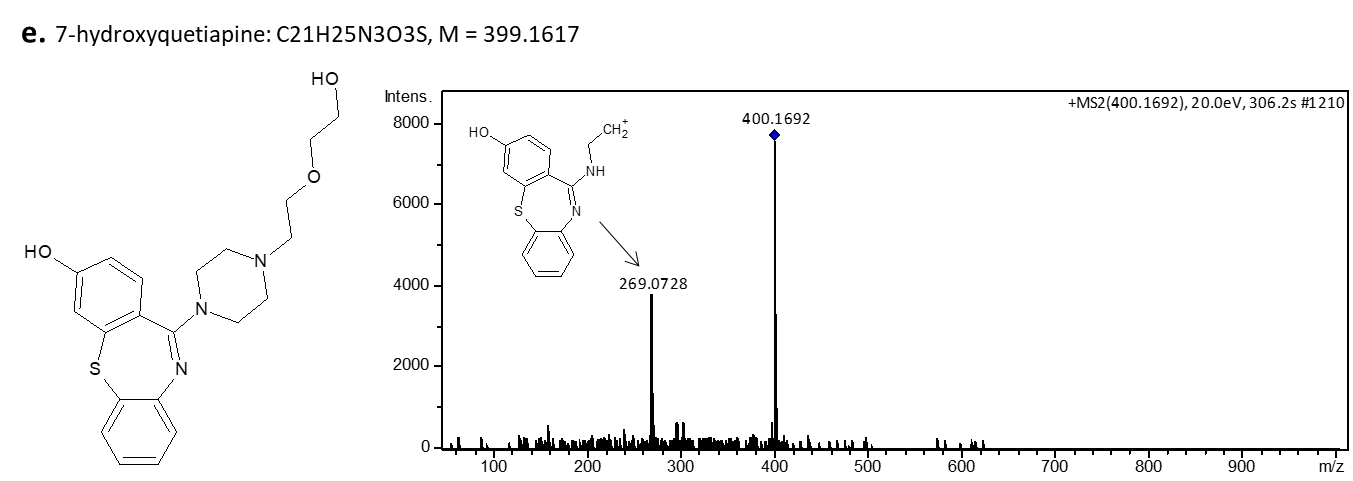


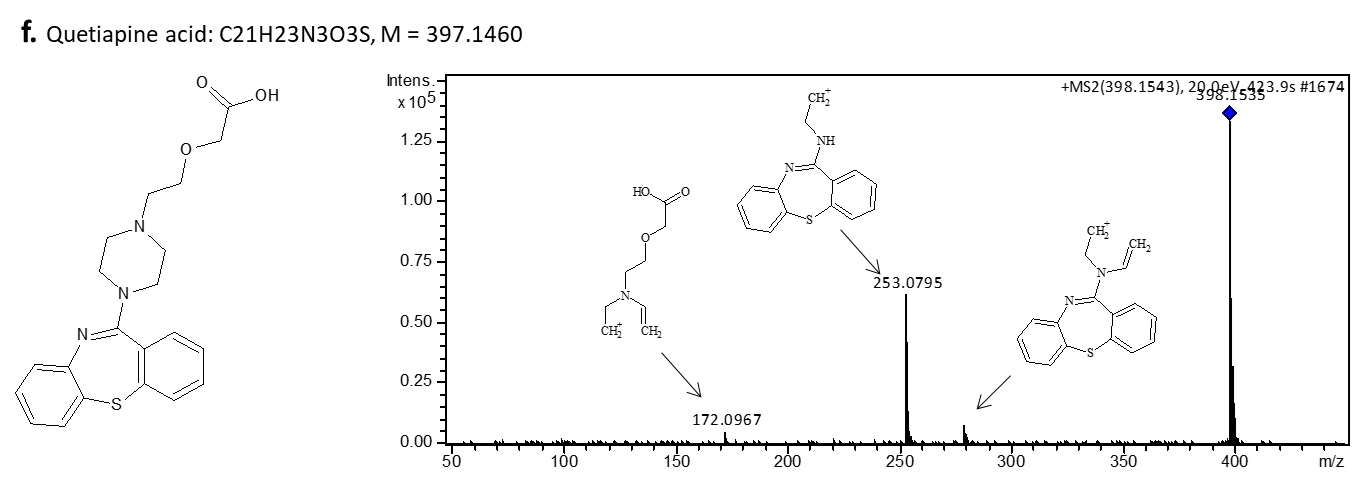


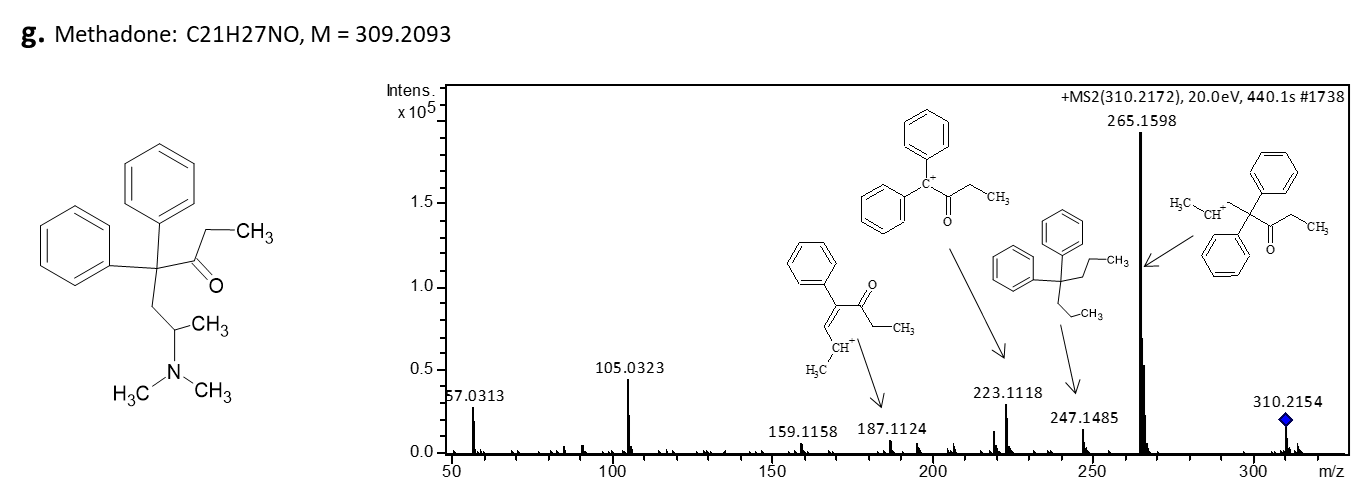


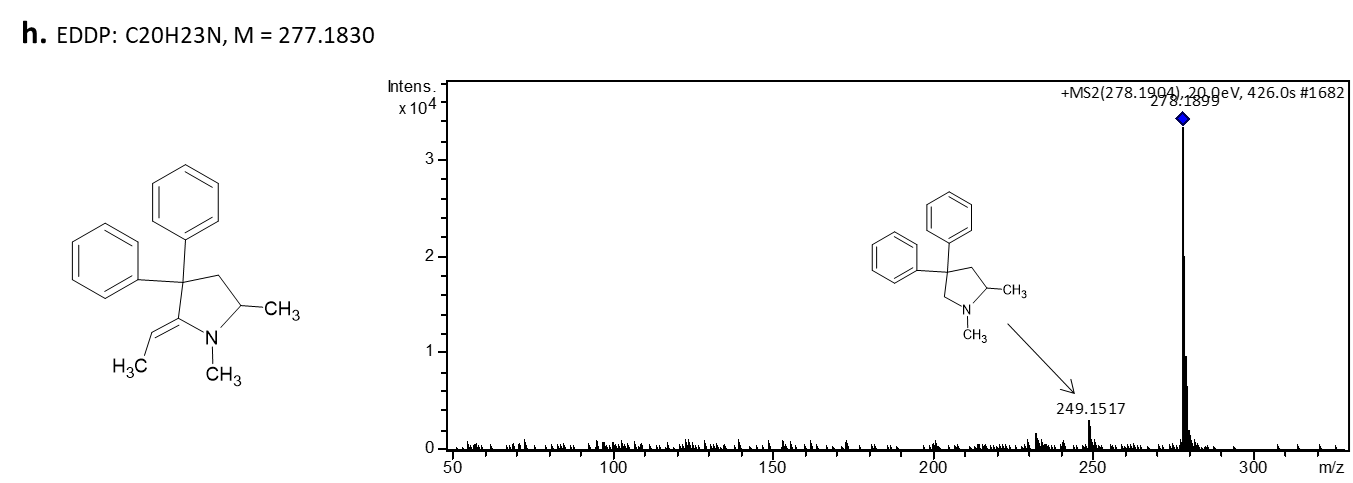


**Fig. S3** MS2-spectra of quetiapine, methadone and their metabolites detected in rat blood and brain.

**Fig. S4** Quantification ratios of norquetiapine and O-dealkylquetiapine vs. quetiapine in midbrain (a) and PFC (b) of rats. Bars represent mean ratios with standard deviations. norQTP =norquetiapine, O-dealkylQTP = O-dealkylquetiapine. Significant differences are marked with asterisks (**p* < 0.1). Data were log-transformed before statistical significance testing.


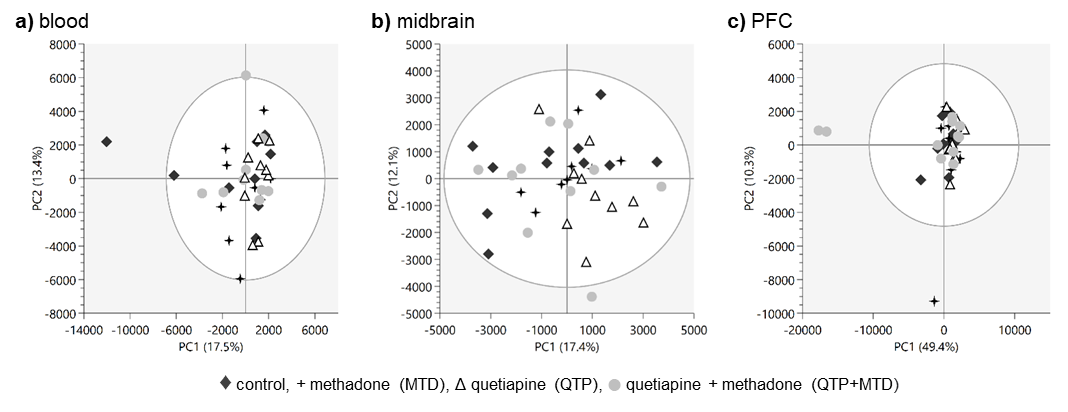


**Fig. S5** PCA scores plots of all features (except the drugs and their associated metabolites) following data filtering, normalization and scaling in blood (a), midbrain (b) and PFC (c) in negative ESI mode. Treatment groups are given as control (♦️), methadone (MTD +), quetiapine (QTP Δ) and quetiapine + methadone (QTP+MTD ⬤).
